# Implementation of the Nursing Associate in the NHS: A Rapid Realist Synthesis to Understand Mechanisms of Integration and Workforce Development

**DOI:** 10.1111/jocn.70154

**Published:** 2025-11-28

**Authors:** Zoe Anchors, Justin Jagosh, Sarah Voss, Nicola Walsh

**Affiliations:** ^1^ Centre for Health and Clinical Research University of the West of England Bristol UK; ^2^ NIHR ARC West Bristol UK

**Keywords:** implementation, nurse roles, Nursing Associates, nursing workforce, realist, Registered Nurses, review, workforce issues

## Abstract

**Aim(s):**

To develop theories about how Nursing Associate (NA) roles are implemented and working within NHS practice: What works, for whom, in what contexts and how?

**Methods:**

Rapid realist synthesis of: (1) empirical and grey literature; (2) realist interviews with stakeholders. Sources were analysed using a realist approach that explored the data for novel or causal insights to generate initial programme theories.

**Results:**

Empirical and grey sources (*n* = 15) and transcripts from stakeholder interviews (*n* = 11) were synthesised which identified three theory areas relating to NA implementation: (1) Scope of NA role: Communication and expectations; (2) Variations to the NA model of working; and (3) Career progression: Entry point, stepping stone and career in itself.

**Conclusion:**

The NA holds the potential to improve nursing workforce stability by encouraging locally based, non‐registered healthcare staff to transition to an NA. However, the lack of collective understanding of the NA scope of practice can cause staff friction. It is unknown whether this friction will reduce over time or if staff divisions will lead to further deterioration of the workforce.

**Implications for the Profession and/or Patient Care:**

Ongoing clear communication regarding NA scope of practice needs to be provided to aid understanding of their supplementary role and its potential contribution to nursing teams.

**Impact:**

This work represents a first step to support both researchers and nursing workforce leaders in furthering knowledge of the impact of integrating NAs in diverse healthcare contexts and to unearth the mechanisms underpinning the success or failure of this new role.

**Reporting Method:**

Realist and meta‐narrative evidence syntheses: Evolving standards.

**Community Inclusion and Engagement (CIE):**

Planning of the research design and interpretation of the results was completed with nurse clinicians with experience in the NA role.

**Summary**

- This paper provides a realist perspective on the importance of supported implementation for nursing associates in NHS practice.

## 1 Background

Since 2017, there have been continuing shortages of registered nurses (RNs) (Buchan et al. 2017) with over 43,000 nurse vacancies currently existing in the NHS in England (NHS Digital 2023). The prospective nursing workforce is also compromised with 2000 fewer nurses expected to graduate in 2025 compared to 2024 (UCAS 2022). These shortages, combined with the increasing patient demand, require a future nursing workforce model that can ensure effective and safe health delivery. One strategy to help build the capacity of the nursing workforce and provide alternative routes into the profession has been the introduction of the Nursing Associate (NA) role (NHS England n.d.‐b). NAs are Band 4 nursing employees, ‘bridging the gap’, between a Healthcare Support Worker (HCSW) (Band 3) and RN (Band 5 and above); they have completed a foundation degree and are regulated by the Nursing and Midwifery Council [NMC] (n.d.‐b). Once qualified, NAs can work across all fields of nursing including Adult, Children's, Mental Health and Learning Disabilities. Practice‐based tasks include clinical procedures such as venepuncture and ECGs; recording clinical observations including blood pressure and temperature, liaising with RNs, reporting patient issues and supporting patients and families. The first cohorts of NAs graduated in 2018/19, and in 2024 there were 10,861 on the NMC Register, with further cohorts graduating annually (Nursing and Midwifery Council n.d.‐a). This role is expected to expand; the NHS Long Term Workforce Plan suggests over 64,000 NAs will be working in the NHS by 2036/37 (NHS England n.d.‐a).

Given the relatively recent introduction of the NA role into the NHS workforce, research exploring its implementation is limited. Much of the evidence has focused on experiences of those training to become NAs, student nursing associates (SNAs), (previously known as trainee nursing associates [TNAs]), or NAs in social care, with key findings suggesting that the challenges are lack of role clarity and poor infrastructure and capacity to support the role (King et al. 2020, 2022, 2023; Dainty et al. 2021; Coghill 2018; Kessler et al. 2022). More positively, NAs in social care were able to perform tasks beyond the remit of a care assistant to support busy RNs, providing new skill mix options for social care employers (Kessler et al. 2022). As a bridging role, the registered and generically trained NA was able to perform tasks and take on responsibilities beyond the remit of the care assistant and delegated with greater confidence by busy RNs. TNAs reported a similar inconsistency in support in primary care along with the lack of opportunity to progress to RN status (King et al. 2023). One study that investigated the NA in secondary care (Lucas et al. 2021) found that although the role would be adaptable in different healthcare settings (e.g., mental health settings) and it could help develop HCSWs, it had limitations with highly acute patients, and the necessary training made the role complex and costly. It was concluded that consideration should be given to the most effective contexts for NA practice and that further evaluation is needed to investigate how the role has embedded over time (Lucas et al. 2021).

Some studies have focused on the association between low nursing staffing and patient mortality rates (Needleman et al. 2011; Fagerström et al. 2018), with evidence to suggest a positive relationship between appropriately planned nursing skill mix and patient safety outcomes (Griffiths et al. 2014); higher registered nurse staffing leads to fewer patient adverse outcomes, including death and infections (Griffiths et al. 2016). However, research has yet to fully explore the impact of the NA in the nursing ratio mix. Recently, concerns over patient safety have been raised in relation to a reduction in the RN workforce and the potential ‘substitution’ of NAs for RNs (Aiken et al. 2017; Griffiths et al. 2019). Role substitution involves transferring responsibilities from one healthcare provider to another, such as from physicians to nurse practitioners or physician assistants (Lovink et al. 2019). Furthermore, it has been argued that the addition of nursing assistants (a role in the Australian nursing structure similar to the NA) may lead to RNs spending less time with patients and therefore limiting opportunities for ongoing monitoring, assessment and evaluation required for effective patient outcomes (Twigg et al. 2016). Globally, similar second‐level nursing roles that vary in title have been introduced in other high‐income countries such as Australia, New Zealand, North America and Canada (Lucas et al. 2021; The King's Fund 2018) and European countries such as Germany and Finland. Internationally, studies have identified role confusion, with blurring between the scope of practice of RNs and second‐level nurses in these other countries (Lavander et al. 2017; Lankshear et al. 2016). On the one hand, some second‐level nurses are devalued and underutilised, whereas others are entrusted with expanded duties leading to inconsistency in role expectations (Lucas et al. 2021). Caution must be exercised when comparing diverse nursing workforce models, as these second‐level nursing roles can differ from the NA role in terms of scope of practice, educational preparation and regulatory frameworks.

Introducing the NA role has the potential to create time for RNs to focus on more complex work; provide a new pathway into RN training; and widen participation in the health workforce (Kessler et al. 2020). Despite NAs being deployed across multiple health and care specialties, little information is available to: support the successful implementation and integration of NAs into the workforce; plan the most appropriate skill mix to deliver high‐quality, safe patient care; or establish the clinical and cost benefits of the role.

## 2 Aim

To synthesise evidence about how NA roles are implemented and working within NHS practice: What works, for whom, in what contexts and how?

## 3 Methods

Realist synthesis methodology was applied to available evidence relating to the implementation of the NA role in the UK context. Realist synthesis is a theory‐driven approach to understanding complex interventions in complex environments (Pawson 2006) and seeks to understand what works, for whom, in what circumstances, how and why (Pawson et al. 2004). It was chosen for this synthesis due to variation in the implementation of NAs in different clinical areas in the NHS and the need to explain how key components (e.g., skill mix, patient type, task type) may work in a variety of ways in different contexts.

Data sources included primary research, grey literature and qualitative interviews with key stakeholders. Literature selection and conducting interviews occurred in parallel. A set of ‘initial programme theories’ (IPTs) was drafted from the data sources in the form of ‘if…then’ statements which revealed key mechanisms. A mechanism in realist terms is defined as ‘how people react or respond to resources’ (Dalkin et al. 2015). These IPTs provide explanatory insight regarding NA implementation across the theory areas. Specifically, they describe how and why the NA role generates certain outcomes; both intended and unintended (Jagosh 2019).

### 3.1 Literature

A series of key word searches were developed in consultation with a Librarian and Information Specialist with NHS expertise. Search terms were designed to be sensitive to the research questions and encompass a variety of ways to describe the NA role. The focus was on UK literature as the role of a NA has substantial international variation. The searches were conducted by the NHS England Knowledge Management Team between September and October, 2023 and included academic and grey literature from a variety of sources including Ovid Medline, Embase, Emcare, CINAHL, NHS England and NHS Employers, Google, Google Scholar and relevant online forums and websites (e.g., Royal College of Nursing, British Medical Association, Health and Care Professions Council). Realist methodology supports the inclusion of non‐research material, which can provide causal insights (Jagosh et al. 2022).

Identified literature was screened by two members of the research team (Z.A., S.V. or N.W.). The lens for screening was broad and any literature that addresses the theory under investigation was scrutinised. All studies met the following inclusion criteria: (a) included TNAs or NAs, (b) reported experiential implementation information rather than a description of the role (c) in England and (d) published in English. Papers were excluded if they specifically investigated the training programme for a TNA. Literature in realist reviews is assessed for richness, scientific rigour and relevance to the research question (Wong et al. 2013; Dada et al. 2023; Data S1) and these criteria are used to identify if a study contributes to a theory and/or tests theory and should therefore be included (Dada et al. 2023).

A third member of the team (S.V. or N.W.) resolved any disagreements in selection. Data extraction was led by Z.A. in Microsoft Word. The following information was extracted from the studies: author, year, country, study aim, nursing role, sample and size, research design, theoretical framework and analysis, key findings and implementation issues.

### 3.2 Stakeholder Interviews

Participants were stakeholders related to the implementation of the NA role in secondary care and were identified via key contacts and internet searches of publicly available contact information. Respondents included staff from the Nursing workforce and Higher Education (e.g., Chief Nurses, Directors of Nursing and Workforce Leads and Educators).

Following institutional ethical approval (Reference number: HAS.23.04.102) potential participants were emailed by the research team with an information sheet explaining the purpose and nature of the research, what taking part would involve and encouraged to contact the research team for further information. Participants returned an e‐consent form or provided verbal consent prior to commencing the interview.

The study team developed a topic guide (see Data S2) based on existing literature and study aims. This guide incorporated questions that explored the implementation of the NA role (e.g., How will the NA role be embedded in your department?), effective outcomes (e.g., What would be effective or valued outcomes of the introduction of the NA in your opinion?) and rival theories/potential negative outcomes of the NA role (e.g., What are possible negative outcomes for the introduction of this NA role?). Participants were interviewed via telephone or video teleconferencing software depending on their preference at a time convenient for them. Prior to starting the interviews, informed consent and permission to record the interviews was confirmed. One author (Z.A.), a non‐clinical Research Fellow, collected the interview data between September and November 2023.

The remote interviews were audio recorded and transcribed by a university approved, GDPR‐compliant transcription service. Transcriptions were checked, reviewed and deidentified by the study team to prevent identification.

### 3.3 Analysis and Synthesis

The literature and interview transcripts were analysed and synthesised to populate IPTs using a realist logic of analysis (Jagosh 2020). This analysis was led by one author with extensive experience in realist methodology (J.J.) who drew together the key insights into a single narrative document. Analysis of the literature provided a framework of ideas around which to build the synthesis, and reflections on the stakeholder interviews were incorporated into this framework. The process was conducted iteratively, in that drafts of the synthesis were shared with the study team and the Study Steering Committee (who were not research participants or respondents) during development to allow feedback and discussion among the team about the concepts being constructed and to ensure the synthesis accurately reflected the data. There was broad consensus among SSC stakeholders regarding the proposed theories and recommendations. Minor differences of opinion, such as terminology distinctions between NA and RN, were thoroughly discussed and amicably resolved.

## 4 Findings

### 4.1 Literature

**FIGURE 1:**
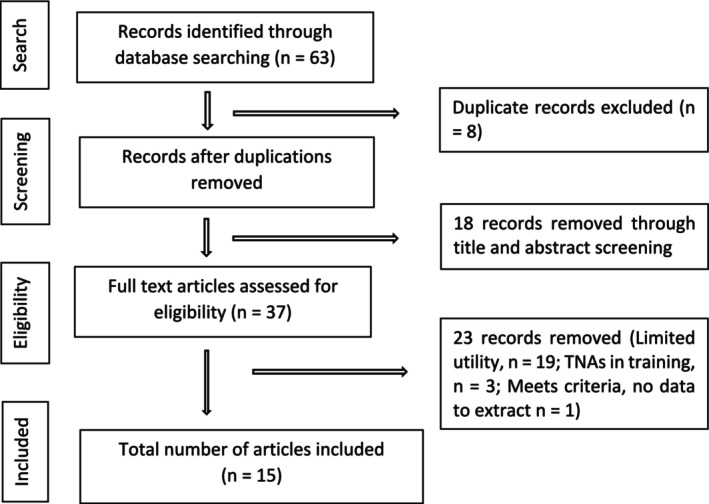
Flowchart of literature searching and screening process.

Records identified in the searches were exported into Covidence software (Covidence Systematic Review Software n.d.) for screening. Figure 1 displays the screening results. From 55 articles, 37 were screened at full‐text level by two reviewers (N.W., Z.A.). A third reviewer (S.V.) discussed and assessed three articles where there was disagreement and consensus was reached. A total of 15 papers met the inclusion criteria. Data extracted from these papers can be found in Table 1. Finally, backward searching was conducted by reviewing the reference lists of included studies and forward searching, aided by the ‘cited by’ function in Google Scholar, was conducted by reviewing articles that cited included studies. One particularly insightful article (Thurgate and Griggs 2023) which reviewed 19 papers on the role of NAs since implementation was searched to identify any additional sources. Resultantly, one additional empirical source was identified (Kessler et al. 2021) and included.

**TABLE 1:** An overview of the included studies (*n* = 15).

Author	Year	Location	Aim	Role	Sample	Sample size	Design	Theoretical framework and analysis	Key findings	Implementation issues
King et al.	2020	England	Explore TNA motivations, experiences and career aspirations	TNAs	TNAs registered at University in the North of England	Three focus groups (*n* = 15)	Exploratory qualitative	Thematic analysis	TNAs are motivated by affordable, local, career development TNAs rely on broad support networks to build their occupational identity	During training TNAs face challenges relating to clinical support, academic workload and uncertainty about future career opportunities They experience role ambiguity both individually and across the wider organisation Some TNAs perceived that their nurse colleagues felt threatened by the new NA role
Giddings	2020	England	Editorial—An NA describing their experiences as a TNA and NA	NA	N/A	N/A	N/A	N/A	Editorial – example of individual experiences included: – ‘Did not get the protected time to learn’ – ‘Completely out of my depth and overwhelmed by the ward and what individuals' expectations were of me, or what other professionals' expectations were of me’! – ‘Colleagues that I worked with did not know what a nursing associate was’	N/A
Dainty et al.	2021	England	Capture the lived experience of some of the first TNAs	TNAs	Some of the first trainee nursing associates (TNAs) during the pilot of the role	4 interviews	Qualitative	Interpretive phenomenological analysis	Six main themes: challenges relating to training; developing new skills; opportunity; the importance of support; the impact of the role; and understanding the role	A lack of knowledge about their role within clinical settings, and that this had a significant impact on them and the way that they were initially received in practice
Lucas et al.	2020	England	Establish the views of a range of stakeholders about their experiences of the newly implemented NA in England and its potential to contribute to patient care	NA in secondary care	Healthcare professionals in acute secondary care over 4 hospital sites – nursing directors, ward managers, nursing associates	33 interviews and 1 focus group	Qualitative descriptive	Consolidated Framework for Implementation Research (CFIR). Data analysis was initially deductive, using thematic framework analysis	Role was broadly adaptable to different healthcare settings Provided a positive professional development mechanism for healthcare support workers Training commitments made implementing the role complex and costly. Role had limitations, particularly with intravenous medicine management	Implementation impeded by rapid pace and consequent lack of clear communication and planning Training complex and expensive Limits with high‐acuity patients and intravenous medicine
Kessler et al.	2022	England	Evaluating the introduction of the NA in social care	NA in social care	Stakeholders in HEE, Support organisations, Care homes and T/NAs	34 interviews	Qualitative scoping		Diverse range of challenges to the introduction of the NA role in social care: – Dedicated roles: Dedicated roles to support TNA programmes have been key to the development of the role – Wider agendas: Although not raised directly in many of the interviews the development of NAs in social care could contribute to wider policy commitments to developing skills and tackling inequalities	Training infrastructure: A fragmented model of service provision rendered it difficult to establish the training infrastructure needed to support the role Financial support: Questions raised about whether there is scope for supportive funding and also cost implications from the pay rises NAs might expect to see following qualification and registration Clinical Commissioning: As the role contributes to the upskilling of the social care workforce, issues of financial return to the provider for this care emerged Local authorities and partnerships: Local authorities have been seen to play a key role in the development of the role. However, involvement of council adult social care departments in the development of the role has been limited
Joyce‐McCoach et al.	2023	International	Explore what is known about transition and pathway programmes from second to first level nursing	2nd level nurses	N/A	Articles between 2000 and 2022	Scoping review	Informed by the Joanna Briggs Institute approach	Transition programmes are often undertaken to open up career pathways, job and financial advancement	Programmes can be challenging as students seek to maintain dual identities, grapple with academic requirements and juggle work, study and personal demands. Despite their prior experience, there is a need for students to receive support as they adjust to their new role and scope of practice
King et al.	2022	England	Understand the factors that influence career choices of trainee nursing associates	TNAs	TNAs	Interviews 2020 (*N* = 14) and 2021 (*N* = 13) Diary data	Longitudinal qualitative study	Thematic analysis	Nursing associate training was viewed by some as a bridge into registered nursing Those preferring to remain as nursing associates were keen to embed the bridging role between healthcare assistants and Registered Nurses, valuing a positive workplace culture	Role ambiguity led several to seek perceived security offered by the Registered Nurse profession
Kessler et al.	2020	England	Evaluating the Introduction of the NA in the Livewell Southwest	T/NAs	Stakeholders: Senior managers, NAs, TNAs and NA line managers	8 Focus groups 3 interviews	Qualitative	Not provided	Three main themes: – Rationale, for example, role develops new career pathway and assists with ‘grown your own’ nursing workforce – Process, for example, Strong support for the role such as a dedicated Place and Development facilitator for the role – Impact, for example, role enriched rather than diluted the skill mix	Lack of role clarity The rapid desired progression of TNAs moving to RNs risked weakening the establishment of a registered bridging role in the organisation and the capacity of qualified NAs to consolidate their skills
Thurgate & Griggs	2023	England	Reviews the empirical research evidence relating to the NA since its implementation in England in 2017	NA	N/A	Articles between 2017 and 2022	Review	Thematic analysis	Nineteen papers with 6 themes: lack of support from others; career development; organisational readiness; resilience in the face of adversity; cost; and worker and learner identity	Organisational readiness, poor status and recognition as a learner Poor awareness among staff of the NA role
Fewings et al.	2022	Not provided	Explore the role identity of the TNA and qualified nursing associates, following a systematic review of the available literature	NA	N/A	Articles between 2015 and 2020 on NA role	Systematic review	N/A	Six articles included: – Value that the role of the TNA can have on patient careTNAs can provide a diverse workforce that can work across different clinical settings and can support areas experiencing staffing shortages – TNAs can deliver the more hands‐on care elements – TNAs can ‘fill the gap’ between the unregistered workforce and the registered nurses – TNAs felt in some instances that they had a direct impact on health outcomes for patients	Lack of understanding of their role in the areas they are working TNA unsure of their role and found it difficult to distinguish their previous healthcare assistance role with the expectations of the new TNA role Open hostility towards TNAs, who were viewed by registered nurses as threatening their own jobs and status as a cheaper alternative
Coghill	2018 (part 1)	England	Early‐experience evaluation of TNA roles, focusing on how the TNAs balance being a ‘worker’ and a ‘learner’ in clinical practice—Part 1	TNAs	TNA roles across the northeast of England	WP1: scoping exercise across the regio; review of demographic data and placement models; Survey of TNAs (*n* = −39) WP2: 4 focus groups with TNAs; Survey of nursing leads (*n* = 8); 3 focus groups with mentors and managers	Mixed methods	Not provided	Details are described in paper (Part 2) below	Details are described in paper (Part 2) below
Coghill	2018 (part 2)	England	Explain in detail the findings from Part 1 above	TNAs	TNA roles across the northeast of England	As above	As above	Not provided	Role clarity, placement models, mentorship and protected learning time were all influential factors	Role clarity: how to raise the profile of the TNA role and share this with staff and the public Debate: are the TNAs ‘learners’ or ‘workers’? Being a worker gives the TNAs a sense of belonging, but also need to look at the learning opportunities Mentorship: issues with having that time out with your mentor and the increasing demand on the number of learners that require mentors Placement models: the block placement model was the preferred Difficulty uncoupling their previous HCA role with their TNA role
King et al.	2023	England	Explore the experiences and career development opportunities for TNAs based in primary care	TNAs in primary care	TNAs in primary care	11 interviews	Qualitative exploratory	Thematic analysis	Four key themes: – Valuable opportunity for career progression – Secondary care emphasis of NA training – Inconsistency of support – Constraints on learning and development opportunities	TNAs frustrated by the ‘emphasis on secondary care’ in both academic content and placement portfolio requirements Felt that their primary care managers did not fully appreciate the time commitment or supervision required of the programme Constraints included the opportunity to progress to become registered nurses
Kessler et al.	2020a	England	Evaluating the Introduction of the NA in the Cambridgeshire and Peterborough NHS Foundation Trust	NA	NAs and programme managers	14 interviews	Qualitative	Not provided	Three main themes: – Rationale, for example, NA programme principally driven by the career aspirations of particular individuals, rather than the service development and design needs of the Trust – Process, for example, Strong support for the role such as a dedicated CPFT established a new dedicated role to support and manage the NA programme – Consequences, for example, a universal desire among NA interviewees to become an RN	With the regulation of the NA role by the NMC ongoing at the time of training, there were uncertainties and disruptions, affecting in particular the curriculum and the NA interface with the HEI Progression to registered nursing was problematic for some NAs (e.g., uncertainty whether a Band 5 nurse post would be available). Considerable uncertainty at team level about the nature of the new role even as the NAs ‘hit the ground’ after 2 years training
Kessler et al.	2021b	England	Informs who T/NAs are, what they do, how they experience employment or training and how they view their working lives.	T/NAs	T/NAs	*n* = 516	Survey	Descriptive analysis	Reports extensive detail on the following: – Background, for example, demographic profile of TNAs and NAs is very similar – Work patterns, for example, Most T/NAs have progressed to the role from an HCA post with their current employer – Training, for example, the most common training model was one founded on hub and spoke arrangements – Work experience, for example, Understanding of the new role among the T/NAs work colleagues remained limited	There were clinical settings stubbornly resistant to the NA role, for example, outpatients' departments and operating theatres. Training was challenging for TNAs, for example, completing formal assignments, and on the pressures faced in seeking to deal with competing demands of work, home and college. Some NAs noted some unevenness in whether they were counted in the registered or unregistered numbers, prompting concerns among them as to whether their skills were being fully utilised.

Abbreviations: NAs, nursing associates; NMC, The Nursing and Midwifery Council; TNAs, training nursing associates; WP, work package.

### 4.2 Stakeholder Interviews

Eleven semi‐structured interviews with 12 participants were conducted and lasted an average of 40 min (ranging from 27 to 56 min). Participants included Directors or Assistant Directors of Nursing (*n* = 6); Clinical Nurse Educators (*n* = 3), Chief Nursing Officers (*n* = 2) and an academic (*n* = 1). Participant characteristics (gender and specific job title) are not provided as this would potentially lead to their identification.

### 4.3 Synthesis

Synthesis of the review and interview data identified three areas, supported by a total of nine IPTs. These were: (1) Scope of practice: Communication and expectations; (2) Variations to the NA model of working; and (3) Career progression: Entry point, stepping stone and career in itself. Each theory area is discussed below with key literature references or stakeholder quotes and generated ‘if’, ‘then’ statements.

#### 4.3.1 Scope of NA Role: Communication and Expectations

Clear communication about the NA role was key to successful implementation. Adequate explanation about the role assisted RN staff to adjust and understand how NAs can fit within existing workflow and staff configuration. In some cases, poor communication led to resistance and hostility from RNs and other staff, resulting in NAs feeling confused about their role, and how and where they fit into clinical teams (Fewings et al. 2022, 481). Explicit competencies associated with the role were helpful in educating all relevant staff about the scope:

Our nursing associate manager…she pulled together this competency pack, which has been used across our ICB, and she spent a lot of hours developing it and together, we look at how we could support staff. And I think that's a transferable product, in order to support others. (Participant 1)

Widespread confusion regarding the NA scope of practice was also reported in different settings, in particular how it differed from the RN role (Joyce‐McCoach et al. 2023, 14). Some interviewees emphasised the need to specify NA scope of practice because it manifested differently in different workplace settings and from speciality to specialty:

We have a terrible habit of not thinking wider than the organisation we work in, but these posts exist across the whole country. So, surely there must be generic, let's say if you wanted to work in endoscopy as a nursing associate there must be generic endoscopy scope of practice, and it just feels too difficult to get to. So, it would be great if that were easily available to everybody. (Participant 5)

This confusion impacted the NAs' professional identity with consequences for their motivation and self‐purpose:

If you don't have an identity in your role, you question as to your…purpose that you're there and therefore the value and that's going to affect your motivation. If you just feel like you're plus‐one or that you're not recognised and appreciated for the knowledge, skills and experience that you have, to the level at which you should, you feel you should be. Then you're either, then you're going to give up in one way, shape or form…So, we have an obligation to our workforce… to make sure that we, when we bring in any new role. That we land it properly. (Participant 8)

It was important for NAs and other staff to understand the NA scope of practice and what it meant for the NA to work at the top of their scope but not beyond. Some nurse leads expressed interest in exploring and expanding the NA scope of practice to the broadest extent possible within the boundaries of the professional regulations:

We've certainly got a growing number of Registered Nursing Associates across the patch, and that's interesting, because we're now testing some of those specialist competencies, that aren't in that initial framework, that were developed, but actually, we recognise that as this group of staff develop their skills but also as we develop our confidence around the role, we can expand their scope of practice. (Participant 1)

Other nurse leads were deliberately limiting the NAs to low‐risk universal tasks, even when there was room for expansion within the registration to do more, to avoid role blurring and consequent potential friction with RNs who may feel threatened by NA role creep.

The progression of skill acquisition of the NA over time is likely to result in role blurring. It was important to maintain distinction between NA and RN roles:

I think most importantly it is being very clear with teams in terms of the differences of the Registered Nurse and the Registered Nursing Associate. Because what will tend to happen is the longer they are embedded in post the less distinct that difference will become, because they will start to take on extended roles such as IV administration, and all sorts of different things. So how do you then continue to determine the difference between the two roles because then you will come into ‘why should I do that if they are getting paid to be a Band up’. (Participant 5)

Indeed, some NAs expressed the belief they were ‘doing everything the RN is doing’ while others felt intimidated at the prospect that staff expected them to do everything the RN does. These factors have contributed to some extent to negative work environments involving pushing and pulling from a mismatch in expectations about the role and scope of practice in relation to already existing roles:

I felt completely out of my depth and overwhelmed by the ward and what individuals' expectations were of me, or what other professionals' expectations were of me. To be honest, I did not really know what my own expectations were, but I felt overwhelmed with anxiety when it was assumed I was a registered nurse and knew what to do. (Giddings 2020, 403)

One unintended consequence of the inclusion of the NA role could therefore be workforce instability. RNs potentially felt undervalued and threatened by role creep of NAs and leave the profession due to diminished work satisfaction:

Now, all of a sudden we're saying that, well, actually a Band four registered nursing associate can look after the same patient, as a band five Registered nurse…we end up back to where we were, twenty, thirty years ago. Well, why do you need to have a degree to look after an ICU patient, because your registered nursing associates don't? and, it just feels like a step on the road to de‐professionalisation of nursing. (Participant 11)

**1. If…then Statements revealing key mechanisms**

*(1.1) If RN staff receive clear communication clarifying the NA role scope and how it is different from the RN role, then RNs will not feel that the NA role constitutes role substitution (mechanism), which will improve NA integration into the workforce*.

*(1.2) If communication is inadequate, and RNs perceive that it is about role substitution, this will lead to increased resentment of NAs (mechanism), causing friction in the workplace, leading to attrition of both of NAs and RNs*.

*(1.3) If NAs are to maximise their scope of practice, RN staff may be hostile (mechanism), leading to attrition of both RNs and NAs. Alternatively, if leadership encourages this, NA staff will realise their capabilities (mechanism) which will enhance the skill mix*.

*(1.4) If nurse managers are concerned about friction with role expansion, they will deliberately limit the NA to universal tasks even if the NA can do more. This may reduce friction within the team, but NAs may feel frustrated (mechanism) in their role leading to disengagement or attrition*.

#### 4.3.2 Variations to the NA Model of Working

The data suggested that NAs were being integrated differently in different healthcare settings with accountability implications. Two examples of workflow models were described, both involving RN supervision of the NA:

- NA patient allocations with supervision: RNs and NAs work in a ‘partnership‐style’, each having allocated patients, each being accountable for their patients, with NAs following prescribed functions and being supervised by the RNs as needed.
- RN‐exclusive allocations with NA support: All patients were allocated to the RNs only, with NAs maintaining a supplementary role, assisting the RNs with allocated tasks.

One interviewee described model (a) as follows:

Potentially you could have a registered health care support worker, or a registered nurse associate working, in partnership with a registered nurse, looking after two ICU patients. (Participant 6)

Alternatively, the data showed the second model in which RNs and NAs were in a more traditional hierarchy (rather than partnership) in which the NAs were treated similar to non‐registered healthcare assistants to support the RN in the supervision of all patients. This model required a fair amount of ‘situational decision‐making’, (Participant 9) meaning that managers needed to increase their responsiveness to allocating tasks to the NA in a dynamic context of critical patient needs. In this approach to NA integration, RNs also expressed concern about how many people they will need to supervise instead of work alongside of:

That was part of the concern raised, from a registered nurse perspective, well how many people am I going to have to countersign and oversee, is it going to be more work for me? (Participant 2)

Others suggested that for deteriorating patients, a more proactive and holistic approach to care needed to be utilised:

People don't stay static, they'll often go up and down within a 12‐hour shift. So, the leader of that shift needs to be really mindful about where is the nurse, the nursing associate, the healthcare assistant. So, the leadership within the service has to be really confident that they're putting the right resource in the right place. (Participant 9)

There were objections by some interviewees to the idea that NAs could supervise patients in a partnership style alongside RNs, citing the difference as delivering ‘prescribed care’ over assessment capabilities and who was accountable to the patient:

A registered nurse associate can deliver prescribed care whereas a registered nurse can review, assess, and manage care so that, if there was a change in care they would deliver that…and the registered nurse would have to oversee that signing of that care provision by the NA, and so how do we get that accountability right? (Participant 2)

Some interviewees reported that having the NAs engage in prescribed care (and not assessment) meant that there was more time for the RN to engage in holistic care, something that was recognised as beneficial in the earlier State Enrolled Nurse (SEN) role and could be a benefit of the NA role:

We're seeing less supported time for registered nurses to sit and develop a relationship with patients and families, which used to be…the cornerstone of nursing and how you developed bespoke care plans, packages for patients by getting to know them…And that whole ‘care’ element, we're seeing more and more concerns raised by families and patients themselves, in terms of communication, a lack of care, a lack of understanding of patient's, of their individual needs. And you know, what was being described, what was missing was the old SEN. (Participant 8)

On the other hand, some suggested that allocating tasks to NAs prevented the RNs from having a holistic approach because it created ‘distance’ from the patients, and the potential to miss assessment opportunities (e.g., washing the patient to determine their level of self‐care, Participant 12).

The data also suggested that the policy on staff ratios should be discussed when considering NA integration. Some participants reported that the NA role works best in larger workforce settings in which there is a lower staff‐to‐patient ratio and enough RNs to complete assessments to which NAs can follow with prescribed care:

So when you've got a small ward, that functions on two, maybe three registered nurses per shift, it's, it becomes, well if there's only two registered nurses per shift you can't count them as a registered practitioner. You still have to have two registered nurses on the ward. They become one of the non‐registered workforce. (Participant 8)

Regarding ratios however, some suggested that safety was mainly ensured through clarity of role scope and optimization of the skill mix:

You can easily put an NA in on every shift, on every ward, and not water down the sort of national guidance. But the key to it all, I think is in that having the clear vision of what the people who are doing the role, what they can and cannot do…having that clear in your mind so that you can build your skill mixes around NAs being in the numbers, but giving you enough flexibility to keep it safe. (Participant 7)

**2. If…then Statements revealing key mechanisms**

*(2.1) If NAs have an allocation of patients and their RN supervisor is not available nor is directly assessing the patient, the NA will lack the confidence (mechanism), to observe and care for the patient in a safe manner*.

*(2.2) If patients are allocated exclusively to the RN, they will receive ongoing attention by a staff member who is capable of appropriate care when needed. However, such a model may lead to NAs feeling dissatisfied (mechanism) with the delegation of less complex tasks*.

#### 4.3.3 Career Progression: Entry Point, Stepping Stone and Career in Itself

The findings suggested that the NA role allows people who are not university‐oriented or are from socially disadvantaged groups to enter into a healthcare profession through a more vocational route:

For those that aren't able to access university, at any particular point in their lives, it gives them an opportunity they wouldn't have. Which I think, as a city centre hospital, we've got a lot of social deprivation in our local community is an important factor. (Participant 7)

Many NAs see the role as a stepping stone to university‐level study as a nurse trainee, at a time when they would never have imagined they would quality for a university degree. “I always had this idea of doing it (nursing), but it's like” no, that's something that clever people do, but then, you just apply for it and then, you end up there. (Dainty et al. 2021, 287)

Evidence pointed to how times have changed and there were fewer young people wanting to progress through formal university‐based nursing undergraduate degrees and thus the NA role acts as a stepping stone:

We've got a different generation now… And the way that they expect to be taught and educated and employed. And when you play in the whole economic side of things, you know, it's becoming harder and harder to go to university and rack up £30,000, £40,000 debt. (Participant 9)

Related to this was the suggestion that the NA role creates workforce stability by making career progression opportunities available to the locally‐based non‐registered healthcare support staff who transition to the NA role and stay embedded in the same healthcare context; this idea which was explained in one paper as “grow your own nurse” (Kessler et al. 2021).

As a registered role sitting between the care assistant and the Registered Nurse, the NA is a new profession with an extended scope of practice. By offering a new career rung, such a role is viewed as encouraging existing employees to stay with their current employer, while enticing new employees into entry level posts, with the promise of career progression. (Kessler et al. 2022, 14)

The data indicated that the drivers for the NA role as a stepping stone to RN training were due to the desire for career progression, or escaping the challenges associated with the NA role:

It [NA role] provides that career opportunity, should you wish to go further, up and beyond into your registered nursing post. (Participant 2)

Others talked about choosing to undertake an RN “top‐up” programme to escape the challenges associated with the NA role. They described experiences of role ambiguity and conflict and felt that these problems would be overcome by joining the more established registered nursing profession. (King et al. 2022, 2489)

Promoting the idea that the NA role was a stepping stone to becoming an RN was also seen as a way to deescalate tensions and perceived threats of role substitution with RNs (King et al. 2020, 8). As reported earlier, RNs that supervise NAs may intentionally limit their scope of practice to ensure clear demarcation between NA and RN role scope, which may frustrate NAs who feel they are competent to do more and learn through practice. Such frustrations on the part of NAs may push them to become RNs. On the other hand, some have suggested that NAs prefer to remain not to progress to RN as a result of ‘making a difference’ in their current NA role (King et al. 2022, 2491).

**3. If…then Statements revealing key mechanisms**

*(3.1) If the NA has the opportunity to ‘try out’ what it feels like to work in nursing, they will realise their interest (mechanism) in going to studying nursing in a way that would not transpire without the practicum opportunity that the NA role allows*.

*(3.2) If solely as a ‘stepping stone’, NAs will not be taken seriously, and their scope of practice not developed. Whereas if seen as a stepping stone (mechanism) for some NAs, but not all, then the NA role will be developed more fully and better integrated*.

*(3.3) If the NA experiences job satisfaction, they may not be interested in becoming an RN (mechanism), provided there are adequate opportunities for professional growth in the NA profession, which will still be limited in terms of pay and role scope over the long‐term*.

## 5 Discussion

The aim of this rapid realist synthesis was to improve understanding of how, why and in what context NAs are working within NHS practice. Since this is a relatively new role, evidence of its implementation is limited. Given the large numbers of NAs expected to be working across multiple NHS specialities in the near future (NHS England n.d.‐a), the role and its integration deserve explicit attention in terms of how it can both effectively and safely be established. We completed a realist synthesis that included both empirical data and stakeholder interviews. Our findings revealed that the scope of practice, differing ways of working and career progression were all key insights when considering the implementation of the role.

Scope of practice can be defined as the range of roles, functions, responsibilities and activities that the NA is educated and authorized to perform. Despite the provision of standards of proficiency for NAs and RNs (Nursing and Midwifery Council [Great Britain] 2018), the scope of practice for this new role was not clearly differentiated from that of an RN, for NAs themselves and within the wider nursing workforce. Limited understanding of role descriptions and role creep have both been previously reported with new and emerging roles being inserted into primary care (Baird et al. 2022; Jones et al. 2024). The lack of role clarity and poorly defined scope of practice can jeopardise teamwork, particularly in healthcare (Cantril et al. 2019). It can also open staff to potential exploitation where staff work beyond their scope of practice; evidence of this has been found with physician associates (PAs) who report resultant reduced job satisfaction (Ritsema and Roberts 2016).

Our analysis suggested that communication and mixed expectations of the scope of practice led to both intended and unintended outcomes. Clear communication about the role will avoid the RN viewing the role as a substitute for their own role and prevent resulting friction between staff. However, in some cases nursing managers may take this one step further and limit rather than explore the scope of practice of the NA to avoid role blurring and the perception of role substitution. Indeed, the idea of role substitution has raised concerns where inappropriate extensions of the role will create significant concerns for standards of care and increase the risk to patient safety (The Queens Nursing Institute 2024). These concerns also exist for similar roles such as PAs and as a result the British Medical Association (BMA) has set out a safe scope of practice, stating that PAs should not make independent treatment decisions or see undifferentiated patients (British Medical Association 2024). Such strategies around clear communication on the scope of practice would lead to more clarity in the nursing workforce with potential for reduction in staff friction and resentment. However, an unintended consequence of limiting the NA scope would be frustrated staff who would be unable to explore their capabilities which should only serve to enhance the skill mix in nursing. Increased frustration may lead to NA attrition.

As a result of the lack of clarity with the NA scope of practice, variation in how the role works in practice can occur (Fothergill et al. 2022). Such variation of practice has previously been reported within other primary care roles (paramedics; Stott et al. 2024) and secondary care (advanced nurse practitioner and physician associate roles; Wang et al. 2022). We identified two different models of working, one with the RN and NA working in partnership, with NA supervision as required, and the other with the NA as a supplementary role, supporting the RN as directed. Both models potentially offer positive and negative outcomes. In the first model, if adequate supervision is provided to the NA, this would serve to address the increasing patient demand and provide high‐quality care. If the supervision is not sufficient however, the patient may not receive quality care. In the second model, patients are more likely to receive a higher level of care from an RN; however there is the possibility of NAs feeling limited and frustrated and the RN not being freed up from their own workloads. The context of the health specialty would also clearly play a role here. For example, for high‐acuity patients in critical care wards, the care is in a continual stage of flux, involving challenging environments and symptoms that could potentially be beyond the scope of the NA role.

Career progression was found to be a significant factor in the implementation of the NA role. We found clear evidence of the ‘home grown’ applicant (Kessler et al. 2021) being motivated to apply for this new role, along with attracting a diverse workforce outside of the typical university applicant. Such differing life and work experience should certainly help to strengthen the nursing skill mix moving forward. Progression to RN was also evident, with many viewing the NA as a stepping stone to this new career. This is a significant improvement compared to the former (now abolished) state enrolled nurse (SEN) in the UK, which was reported as having limited career prospects (Dan Mason Research Committee 1962). It is possible that NHS Trusts face a potential risk of losing experienced NAs to RN training, which can inadvertently undermine the bridging role that NAs were designed to fulfil. To mitigate this risk, it is essential for healthcare organisations to strengthen the identity and value of the NA role by offering adequate support within the role and tailored recognition that supports long‐term career fulfilment within the NA pathway (Thurgate and Griggs 2023). However, we also identified NAs who wish to remain in the role without an intention to progress to an RN. It will be a challenge to have this role both as a stepping stone and as a career in itself without the development or extension of the scope of its practice. Such an extension should be cautious to avoid the impacts of role blurring.

### 5.1 Strengths and Limitations of the Work

To our knowledge, this is the first realist synthesis of evidence surrounding the NA role. The use of the realist approach is a clear strength of this work as it is highly appropriate to assess the introduction of the role in a rapidly changing healthcare environment where unpredictable forces (e.g., patients' clinical needs, nursing staff shortages) determine outcomes (Jagosh et al. 2022). Guided by realist logic, data were selected based on the relevance and richness of detail to address NA implementation and not by hierarchies of methodology. Furthermore, results from academics experienced in realist methodology were guided by feedback from a Study Steering Committee who has a wealth of clinical and workforce development experience in nursing. Therefore, the theories are resulting from in‐depth, reflective discussions within and between the project team and the stakeholder group, rather than isolated data analysis. This realist synthesis has now theorized these forces and provides a theoretical basis for researchers to investigate the implementation of the NA role. This work represents a first step to support the field in furthering our collective knowledge of the contexts and mechanisms underpinning the success (or otherwise) of such a new role.

This work has one key limitation. Given that the NA is relatively new in the workforce, there were few studies on the impact of qualified NAs in practice, with many studies focusing on the training programme. However, to counteract this, the literature was complemented with a rich and diverse set of stakeholder interviews, allowing for robust theory development. These interviews were still limited by the NA's short time in practice and consequently, the theories put forward should be treated with caution and require empirical scrutinization through future research using primary data to lead to more actionable CMOs.

### 5.2 Recommendations for Further Research

After refining the IPTs (i.e., exploring any new literature and using this evidence to strengthen the theory), we recommend future research testing them in a wide range of health specialties (e.g., critical care, emergency care) where NAs are being deployed. It will be important to analyze how different models of NA working are impacting team working, clinical outcomes and patient safety. Little is known about how NAs contribute to patient safety. There is mixed evidence to suggest that both low and high NA staffing are associated with increased mortality (Griffiths et al. 2019). Providing direct recommendations on nursing staff ratios is therefore required but complex, and we currently only know that ‘higher’ staffing levels are better than ‘lower’ (Ball and Griffiths 2022). While the direct evidence linking NAs to improved patient outcomes is still emerging, early workforce feedback suggests that they play a supportive and increasingly valued role in enhancing continuity of care and holistic patient support (University Hospitals Plymouth NHS Trust 2021). Isolating the individual impact of an NA is challenging due to their role in a team‐based workforce. However, as their role becomes more established, research using more robust mixed methods to measure direct reductions in readmissions, infections or mortality and improvements in patient‐reported outcome measures is anticipated to grow.

Regardless, staffing ratios should not be considered in isolation and different contexts (e.g., patient type, clinical specialty, skill mix, team dynamics, leadership style and organisational culture) will also contribute to the effectiveness of the NA role. In addition to staff ratio guidance, this study has also highlighted additional questions that require further investigation: How can communication efforts (e.g., Competency assessment packs, placement officers and mentoring programmes) improve role clarity at work, and how many NAs want to remain in their role permanently and how many end up progressing to RN training?

### 5.3 Implications for Policy and Practice

The evidence is clear that ongoing communication and development of the NA role may be needed to create genuine, motivated buy‐in from a wider staff workforce. The role is not necessarily welcomed by all professional groups and these staff need more information to understand both the role and also its potential contribution to healthcare teams. This information may need to be customised for different healthcare contexts (e.g., critical care, pre‐hospital) so staff, including NAs themselves, understand that they are a complementary role rather than a substitution. Processes should be put in place for any concerns or issues to be raised where a NA is perceived to be working outside of their scope of practice or not being able to work fully to their scope. For example, the review and refinement of competency assessments may support greater consistency in expectations across wards or trusts (Whelan 2006). This approach can help mitigate the risk of role creep among NAs and ensure that training and assessment processes remain aligned with appropriate and achievable competencies, thereby facilitating more effective and accountable training. Nevertheless, it is equally important to acknowledge the potential risks of over‐regulation, which may inadvertently constrain the flexibility and professional development of NAs within evolving healthcare environments. Therefore, successful integration and growth is likely to depend on a planned process with senior levels of support, local champions, embedded governance and review processes. With the expected exponential increase in NAs in the next few years, this requires prompt consideration. Finally, while comparisons with nursing assistants in other countries should be approached with caution, our findings have the potential to inform nursing workforce decisions outside of England. Regulation for the role has been approved in the devolved nations (Nursing and Midwifery Council 2024) and similar roles exist in other high‐income countries (Lucas et al. 2021).

### 5.4 Conclusion

Employing additional nursing workforce, such as the NA, to support RNs and HCSWs may mitigate the shortage of nurses and improve patient communication. Many healthcare teams now rely on the contribution of professionals with different expertise to meet the increasingly complex needs of the population. This realist synthesis provides a timely and clear articulation of the importance of scope of practice, variation in working and career progression in the implementation of the NA role. There are challenges associated with introducing new roles in the NHS; they can create confusion and possibly even division in the workforce. This research provides evidence that the NA role can both create workforce stability (e.g., encouraging the ‘home grown’ nurse) and create instability (e.g., staff resentment and friction). It is unknown whether these unintended consequences represent a normalisation process that will stabilise over time, or if the divisions will intensify and lead to further deterioration of the workforce. The theories in this paper therefore require testing in future studies.

## Author Contributions

Zoe Anchors: methodology, resources, investigation, writing original draft and reviewing and editing. Justin Jagosh: formal analysis, writing original draft and reviewing and editing. Sarah Voss and Nicola Walsh: conceptualization, funding acquisition, methodology, project administration, supervision and writing – reviewing and editing.

## Funding

This research was funded by Bristol and Weston Hospitals Charity, NHS England.

## Conflicts of Interest

The authors declare no conflicts of interest.

## Supporting information


**Data S1:** jocn70154‐sup‐0001‐DataS1.docx.

**Data S2:** jocn70154‐sup‐0002‐DataS2.docx.

## Data Availability

The data that support the findings of this study are available on request from the corresponding author. The data are not publicly available due to privacy or ethical restrictions.
