# Supplementary material for: Implementation of the Nursing Associate in the NHS: A Rapid Realist Synthesis to Understand Mechanisms of Integration and Workforce Development
Source: J Clin Nurs. 2025 Nov 28;35(4):1877–96. doi: 10.1111/jocn.70154 (PMC12964514; doi:10.1111/jocn.70154)
Supplement: Supplementary file 2 — Data S2: jocn70154‐sup‐0002‐DataS2.docx. [file JOCN-35-1877-s001.docx]

**Supplementary Material**

**S1.** Interview Topic Guide

**Nursing Associates: Interview Topic Guide**

Context of the interviews: *These interviews will be conducted online with 10 - 15 stakeholders (e.g., Workforce development leads, Nursing directors) related to the Nursing Associate role in acute care. UHBW are seeking to understand how best to embed and implement the NA role in an effective and safe manner. While this document provides a guide to the questions asked in the interviews, it will likely differ depend on the flow of conversation in each interview.*

1. **Thank interviewee for their time, explain the purpose of the interview, confirm consent (audio-record consent if required)**
2. **Start with general questions about their role/job – ask them to describe their role e.g.:**

- What is your job title?
- Tell me about your job in a few sentences
- Time in role

1. **Explore their experience of NA working in healthcare services and drivers/understanding of the role:**

- What is your role specifically in relation to NA? *For example, ‘education/training’; ‘commissioning services’; ‘supporting NA in practice’, ‘developing new models of working’*; *working as NA*
- Interviewees’ perceptions/comments of the role and why NA has been introduced.

**4. Explore NA implementation (facilitators and challenges for effectiveness and safety) at macro (UHBW Trust, NHS acute care), meso (the team, the department, the ward) and micro (the NA, other staff) levels.** *Discussion of each will depend on the interviewee’s specific role.*

Let’s discuss how the NA role will be implemented and its challenges/facilitators

- How will the NA role be embedded in acute care/Trust/your area/dept? PROMPT IF NECESSARY: How good is the fit of the NA role? Do you think it will have to be adapted, if so how? How has/will the role been/be communicated? Is there a good understanding of what the NA will do and the scope of their role? How ready are you/they to introduce the NA?
- How would a RNA and their role be introduced to a patient?
- How does the RNA and RN differ in terms of decision making and delegation of patient care?
- What activities and tasks should a RNA do and what activities and tasks should a RN do?
- What is the role of the RN in terms of support and supervision of the RNA?
- What conditions will make the role effective? Explore ratios.
- What can be done to ensure the role is implemented safely?
- What challenges do you see? How can the people who are less supportive of the role be convinced of its successful impact?

**5. Explore effective outcomes of the NA role**

- What would be effective or valued outcomes of the introduction of the NA in your opinion? PROMPT IF NECESSARY: NA free up higher banded staff time to concentrate on most complex cases; NA create additional capacity; NA may improve clinical / satisfaction outcomes.
- If so, what is the reason for this?
- And what about longer term outcomes? PROMPT IF NECESSARY: Encourage uptake of TNA roles

**6. Explore rival theories/potential negative outcomes of the NA role**

- What are possible negative outcomes for the introduction of this NA role? PROMPT IF NECESSARY: NA is a threat to other staff; NA may compromise patient safety; NA will have poor job satisfaction (for NA and RN)
- What is the reason for this?

**7. Anything else you would like to add**
